# 

**Published:** 1852-02

**Authors:** 


*Buffalo Medical College commencement.* — The annual commencement at this Institution occurs on Wednesday, the 25th inst. The public exercises will take place in the afternoon of that day; of which notice of the place and hour will be given in the daily papers of the city. Addresses will be made on the part of the Faculty, by Professor C. A. Lee, and on the part of the Council of the University, by Hon. James Wadsworth, Mayor of the city. Curators, and all others interested in the Institution, are respectfully invited to be present at the commencement exercises.

The examination of candidates for graduation before such curators as may choose to be present, will take place at the Medical College, on Tuesday, the 24th inst. Curators are invited to meet at the College on that day at 10 A.M.

In consequence of this notice the notification of the curators individually will be omitted.

*Lectures on Microscopy.*—We would invite attention to the advertisement of Dr. Dalton's course of lectures, which may be found on the second page of the cover. The course, it will be perceived, commences on the 26th instant. The lectures are to be given in a commodious apartment at the American Hotel. They are to take place in the afternoons and evenings of the days indicated in the advertisement, so that persons preferring either hour can be accommodated.

